# Safety evaluation of preoperative stent insertion and clinical analysis on comparison of outcomes between preoperative stent insertion and emergency surgery in the treatment of obstructive left-sided colorectal cancer

**DOI:** 10.12669/pjms.36.3.1707

**Published:** 2020

**Authors:** Lijiang Han, Xinjiang Song, Bin Yu, Mingliang Zhou, Liping Zhang, Guogang Sun

**Affiliations:** 1 Lijiang Han, Department of Anal and Intestinal Surgery, Shaoxing Second Hospital, No. 141 Yan’an Road, Shaoxing 312000, Zhejiang Province, P. R. China; 2 Xinjiang Song, Department of Anal and Intestinal Surgery, Shaoxing Second Hospital, No. 141 Yan’an Road, Shaoxing 312000, Zhejiang Province, P. R. China; 3 Bin Yu, Department of Anal and Intestinal Surgery, Shaoxing Second Hospital, No. 141 Yan’an Road, Shaoxing 312000, Zhejiang Province, P. R. China; 4 Mingliang Zhou, Department of Anal and Intestinal Surgery, Shaoxing Second Hospital, No. 141 Yan’an Road, Shaoxing 312000, Zhejiang Province, P. R. China; 5 Liping Zhang, Department of Anal and Intestinal Surgery, Shaoxing Second Hospital, No. 141 Yan’an Road, Shaoxing 312000, Zhejiang Province, P. R. China; 6 Guogang Sun, Department of Anal and Intestinal Surgery, Shaoxing Second Hospital, No. 141 Yan’an Road, Shaoxing 312000, Zhejiang Province, P. R. China

**Keywords:** Obstructive left-sided colorectal cancer, Outcome, Primary anastomosis, Stent decompression

## Abstract

**Objective:**

To evaluate the safety of preoperative stent insertion and compare the short- and long-term outcomes between preoperative stent insertion and emergency surgery in the treatment of obstructive left-sided colorectal cancer.

**Methods:**

The clinical data of 302 patients who underwent surgery for obstructive left-sided colorectal cancer from January 2009 to May 2014 were retrospectively analyzed. They were divided into two groups according to whether to receive stenting for the success rate and complications of stent insertion in colonic lumen by colonoscope, and the number of cases of primary resection and anastomosis, and short-term complications such as incision infection, anastomotic leakage, spleen tear and abdominal abscess as well as mortality and survival rate during hospitalization were compared.

**Results:**

The success rate of endoscopic nitinol alloy memorial stent insertion in colonic lumen was 97.62%, and the overall incidence of complications was 14.5%, of which the incidence of serious complications (perforation, stent migration) was 4.76%. The primary anastomosis rate was significantly higher in the stent insertion group (85.71%) than that in the emergency surgery group (36.24%). The overall complication rate in the stent insertion group (14 cases) was significantly lower than that in the emergency surgery group (102 cases). There was no significant difference between survival curves (P>0.05).

**Conclusion:**

Preoperative stent insertion in colonic lumen by colonoscope for decompression is an ideal auxiliary method in the treatment of obstructive left-sided colorectal cancer, and may increase primary anastomosis rate, avoid neostomy, reduce short-term complications, and improve the long-term survival compared to emergency surgery.

## INTRODUCTION

Colorectal cancer is one of the most common digestive malignancies, including colon cancer and rectal cancer. Its incidence shows an increasing trend year by year, ranking only second to gastric cancer and esophageal cancer in the digestive system tumors,^1^ which is related to environment, heredity and many other carcinogenic factors. Colorectal cancer is mainly manifested with change of stool character and hemafecia in clinic. About 10%-40% of patients with colorectal cancer may suffer from acute obstruction,^2^ mostly being located at the junction of the left colon and rectosigmoid.^3^ Besides, 85% of obstruction patients need emergency surgical treatment, the prognosis of which is much worse than that of selective operation.^4^ The mortality rate of emergency surgery is 15% to 20%, with a variety of complications as high as 45% to 81%, while the mortality rate of selective operation is only 0.9% to 6%.^5^ In recent years, stent insertion in colonic lumen is considered to be safe and effective in the treatment of obstructive colorectal cancer, which as preoperative decompression has been adopted by more and more people, but there are not many follow-ups for the long-term results of this invasive treatment, which is only found in only a few cases.^6^ This study summarized the data of 302 patients with left colorectal cancer complicated with acute intestinal obstruction who received treatments with different methods in our hospital from January 2007 to May 2012, which were analyzed as follows.

## METHODS

The clinical data of 302 patients who underwent surgery for obstructive left colorectal cancer in our hospital from January 2009 to May 2014 were retrospectively analyzed after approved by Ethical Review Board on January 6, 2009. They were divided into two groups according to whether to receive stenting: stent insertion group (n=84), in which the patients received stent insertion in colonic lumen by colonoscope for preoperative decompression. There were 72 patients undergoing primary resection and anastomosis, 7 patients with wide metastasis giving up surgery after stenting, 3 patients receiving selective Hartmann operation and 2 patients with intestinal perforation undergoing emergency Hartmann operation; and emergency surgery group (n=218), in which 79 patients underwent primary resection and anastomosis after emergency decompression and the other 139 patients received emergency Hartmann operation.

### Inclusion criteria

Patients with preoperative abdominal pain, abdominal distension, intestinal obstruction, intestinal dilatation or gas-fluid level observed by radiography; with preoperative CT or colon cancer in or below splenic flexure or rectal cancer more than 10 cm away from the anus margin by colonoscopy diagnosis; without preoperative peritonitis; (4) without receiving preoperative adjuvant radiochemotherapy. TNM staging was used. Patients with incomplete medical records were excluded.^7^

### Preoperative decompression

The patients chose whether to receive stent insertion on their own. Stent insertion was accomplished by two endoscopy doctors. The stent was inserted in colonic lumen by colonoscope: after fibro-colonoscope reached the narrow part of tumor, guide wire was led in through the biopsy channel; then it passed the narrow part under X-ray fluoroscopy. Catheter was inserted through the guide wire and an appropriate amount of contrast agent was injected. Doctors then knew about the position, length and narrow degree of intestinal tumor and whether it was complicated by intestinal perforation, decided the type and model of stent to be used, sent the stent and conveyor to the place 2 cm beyond the narrow area under direct-view colonoscope, making the narrow place in the middle of the stent, and then released the stent after confirming its correct position. After stent insertion, patients started to exhaust and defecate and symptoms of abdominal pain and distension were gradually improved. The patients routinely took intestinal bacteriostatic agent and intestinal lavage solution orally for intestinal cleaning before operation and received radical resection of rectal cancer within two weeks.

### Emergency primary anastomosis operation

Patients under 60 with a good basic state, preoperative serum albumin >35 g/L and hemoglobin >100 g/L without cardiopulmonary diseases were chosen. Colorectal radical resection of primary resection and anastomosis was conducted in the emergency treatment after intraoperative simple decompression or lavage after decompression:

Simple decompression primary resection and anastomosis: during the operation, doctors first dissociated colon and broke mesentery blood vessel with the conventional radical operation method, bared intestinal wall in the place where tumor distal intestinal canal was to be cut off, divided intestinal canal and carried it away from the incision after proximal locking, cut intestinal wall open close to tumor, inserted and fastened screwed pipe with a diameter of 2-3 cm, connected the distal end of screwed pipe with the plastic bag under the operating table and fastened the bag mouth and pushed expanded small intestine to colon successively by hand repeatedly until the enteric cavity was empty. Another section of proximate colon was resected according to radical principles of tumor and then intestinal canal end-to-end or end-to-side anastomosis was conducted.

Intraoperative appendiceal stump coloclysis decompression: steps of dissociating and cutting off intestinal canal and decompression were the same as above. Doctors used bowel forceps to clamp terminal ileum after decompression, inserted and fastened No.12 catheter from appendix root, connected catheter with clean enema bag, used 3-6L normal saline to wash colon, then washed it with 500 ml 0.2% metronidazole, removed catheter and conducted appendectomy. The method of resection and anastomosis was the same as above.

Intraoperative closed intestinal lavage device coloclysis decompression: steps of dissociating and cutting off intestinal canal were the same as above. Doctors inserted and fastened the snakeskin supplied in intestinal lavage device through tumor proximal intestinal canal, imported intestinal lavage duct to caecum through side hole and connected the duct with enema bag. Washing and anastomosis methods were the same as above.

### Emergency Hartmann operation

Patients above 60 with a poor basic state, preoperative serum albumin <35 g/L and hemoglobin <100 g/L underwent emergency Hartmann operation.

### Observation indices

The success rate and complications of stent insertion in colonic lumen by colonoscope were analyzed; the number of cases of primary resection and anastomosis, and short-term complications such as incision infection, anastomotic leakage, spleen laceration and intra-abdominal abscess as well as mortality during hospitalization and survival rate during follow-up were compared between the two groups.

### Statistical analysis

All data were analyzed by SPSS17.0 and subjected to independent samples t-test. The numeration data were subjected to χ^2^ test. P<0.05 was considered statistically significant.

## RESULTS

No significant difference was observed between the two groups in age, sex ratio, tumor location, tumor differentiation and TNM staging (P>0.05) (Table-I). The success rate of nickel-titanium memory alloy stent insertion in colonic lumen by colonoscope was 97.62%, and the overall incidence of complications was 14.5%, of which the incidence of serious complications (perforation, stent displacement) was 4.76%; Two cases of perforation occurred in the early stage of stent insertion who underwent emergency Hartmann operation; 2 patients suffered from re-obstruction for stent displacement who received emergency surgery for primary anastomosis after intraoperative intestinal lavage, without the occurrence of anastomotic leakage (Table-II).

**Table-I:** Clinical pathological characteristics.

Item		Stent insertion (n=84)	Emergency surgery (n=218)	P-value
Age		60.25±12.17	61.03±12.01	>0.05
Gender	Male	50	131	>0.05
	Female	34	87	
Tumor site	Descending colon	32	76	>0.05
	Sigmoid colon	40	113	
	Rectum	12	29	
Tumor differentiation degree	Mucinous adenocarcinoma	2	6	>0.05
	Low	6	25	
	Medium to high	76	187	
TNM stage	II	12	33	>0.05
	III	57	162	
	IV	15	23	

**Table-II:** Stent insertion outcomes.

Complication	Case No.	Percentage (%)	Outcome
Mild abdominal pain	4	4.76	Elective surgery (primary anastomosis)
Mild bleeding	3	3.57	Elective surgery (primary anastomosis)
Rectal tenesmus	1	1.19	Elective surgery (primary anastomosis)
Perforation	2	2.38	Emergency surgery (Hartmann)
Stent migration and re-obstruction	2	2.38	Emergency Surgery (primary anastomosis)
Overall	12	14.29	

In the 84 patients in the stent insertion group, 72 underwent the primary anastomosis, accounting for 85.71%; 4 cases of anastomotic leakage healed after conservative treatment; in the 218 patients in the emergency surgery group, there were 79 cases of primary resection and anastomosis and 13 cases of anastomotic leakage, of which 13 patients healed after conservative treatment, 3 underwent proximal colostomy and 1 died of septic shock. The overall complication rate (two or more complications in some patients) was significantly different between the stent insertion and emergency surgery groups, in which the incision infection cases were significantly higher in the emergency surgery group than those in the stent insertion group, and the cases with other complications such as anastomotic leakage, spleen laceration, intra-abdominal abscess and intestinal obstruction in which surgery was needed, incisional hernia, cardiovascular accident, respiratory failure, catheter-related sepsis in the emergency surgery group were higher than those in the stent insertion group, but no statistically significant difference was found. There was no death in the stent insertion group during hospitalization, while four deaths in the emergency surgery group, of which 1 died of anastomotic leakage combined with septic shock, 2 died of cardiovascular accident and 1 respiratory failure, but there was no statistical difference between the two groups (Table-III).

**Table-III:** Short-term outcomes after surgery.

Item		Stent insertion	Emergency surgery	P-value
Primary anastomosis		72	79	<0.05
Complication	Incisional infection	6	43	<0.05
	Incisional hernia	1	7	>0.05
	Anastomotic leakage	4	17	>0.05
	Intra-abdominal abscess	1	9	>0.05
	Intestinal obstruction needing another surgery	1	7	>0.05
	Spleen laceration	0	6	>0.05
	Catheter-related sepsis	0	4	>0.05
	Cardiovascular accident	0	4	>0.05
	Respiratory failure	1	5	>0.05
Combined complications		14	102	0.000
Death during hospitalization		0	4	>0.05

### Long-term survival rate

In the stent insertion group, 5 patients were lost to follow-up and four patients died of other diseases. The follow-up time was (45.86 ± 14.61) months. In the emergency surgery group, 11 cases were lost to follow-up and six cases died of other diseases. The follow-up time was (44.92 ± 15.27) months. The two year survival rates were similar in the two groups, while the survival rate two years later was slightly higher in the stent insertion group than that in the emergency surgery group, but no significant difference was noted between the two groups (P>0.05) (Table-IV).

**Table-IV:** Long-term outcomes.

Item	Stent insertion (n=84)	Emergency surgery (n=218)
Follow-up (case)	5	11
Died of other diseases (case)	4	6
Follow-up time	45.86±14.61	44.92±15.27
2-Year survival rate	71.43%	70.18%
Number of patients surviving for 2 years and above	60	153

## DISCUSSION

The success rate of nickel-titanium memory alloy stent insertion in colonic lumen by colonoscope is fairly hight,^8^ but there are complications such as perforation, haemorrhage, slight abdominal pain, stent displacement, stent blockage and re-obstruction and serious complication perforation is rarely seen.^9^ The success rate of stent insertion is 97.62% in this study, close to that reported in a previous literature.^10^ Two cases of perforation occurred in the early stage of stent insertion, which no longer occurred later. With the improvement of techniques, perforation complication might be reduced. Two cases of obstruction due to stent displacement were incomplete obstruction. Therefore, enteric stent insertion should be used with caution for patients with incomplete obstruction. Nickel-titanium memory alloy stent insertion in colonic lumen by colonoscope can rapidly eliminate symptoms and X-ray abnormal manifestations of intestinal obstruction,^11^ win sufficient time for bowel preparation for patients that might have radical cure, enable patients to have time for body state adjustment and avoid stress in emergency surgery, increase the opportunity of primary anastomosis in selective operation and reduce complications.^12^ Therefore, primary anastomosis rate of the stent insertion group was significantly higher than that of the emergency surgery group.

Since the 1980s, global literatures have reported colon decompression primary resection and anastomosis with different methods and curative effects.^13^ In this study, emergency surgeries included Hartmann operation, simple decompression primary resection and anastomosis, primary resection and anastomosis of intraoperative appendiceal stump coloclysis decompression and intraoperative closed intestinal lavage device coloclysis decompression according to different statuses of patients. The overall short-term complication rate in the emergency surgery group was significantly higher than that in the stent insertion group. The incision is often larger as decompression is required in emergency surgery, and the incidence rate of postoperative incisional hernia is higher than that of selective operation.^14^ In operation under emergency conditions, patients have a strong reaction of stress, so that cardiopulmonary complications increase relatively. Catheter-related sepsis occurred more easily in the emergency surgery group, which was related to its high incidence of anastomotic leakage. 4 cases of catheter-related sepsis in this group were related to long-time intravenous nutrition. The primary resection and anastomosis rate of the stent insertion group was significantly higher than that of the emergency surgery group, indicating that the incidence rate of anastomotic leakage in the emergency surgery group is higher and severer; patients often have a poor basic state and conservative treatment is not easy to succeed and has a high risk. The emergency surgery group has 4 cases of death during hospitalization, while no death occurred in the stent insertion group, indicating that, in operation under emergency conditions, patients have a poor basic state without enough time for adjustment, as well as strong stress reaction and high incidence of various complications. One patient may have more than one complication and multiple complications may cause the death of patients. Therefore, selective operation is much safer than emergency surgery.

The long-term survival rate of the stent insertion group was slightly higher than that of emergency surgery group. The reason might be that emergency surgery has a poor surgical field exposure; it is very difficult to realize radical cure and intestinal canal squeezing might inevitably cause tumor cells removal and implantation or paradoxical metastasis of tumor embolus; patients undergoing emergency surgery have increased postoperative infective complications and the immune function of body diminishes after operation, creating conditions for tumor recurrence. Anastomotic leakage can increase local recurrence rate, thus causing decrease of long-term survival rate.^15^ Patients have many short-term complications after emergency surgery. Delaying the operation to the first chemotherapy interphase might be another reason causing its low long-term survival rate.^16^ It has previously been reported that the 5-year survival rate after stent operation was lower than that of selective operation.^17^ It is also reported that complications after stent insertion were reduced compared with emergency surgery, but the long-term survival rate did not have significant differences.^18^ For patients that cannot undergo excision, complications of simple stent decompression are lower than those of operative decompression, but their survival rates are similar.^19^ It is worthwhile to conduct further multicenter studies to evaluate the long-term effects of stent insertion.

## CONCLUSION

For primary resection and anastomosis in patients with obstructive left colorectal cancer, preoperative decompression through stent insertion in colonic lumen by colonoscope is an ideal auxiliary method, so as to avoid emergency surgery, increase opportunities of primary resection and anastomosis, avoid neostomy, reduce short-term complications, and improve long-term survival rate to some extent compared with emergency surgery.

### Authors’ Contributions:

**LH & XS:** Study design and significant manuscript revision.

**LH, BY, MZ, LZ & GS:** Manuscript drafting, clinical data collection and analysis.

**LH, XS, BY, MZ, LZ & GS:** Approval of manuscript submission.

**LH, XS, BY, MZ, LZ & GS:** Responsible and accountable for the accuracy or integrity of this work.
